# Utilizing Moist or Dry Swabs for the Sampling of Nasal MRSA Carriers? An *In Vivo* and *In Vitro* Study

**DOI:** 10.1371/journal.pone.0163073

**Published:** 2016-09-14

**Authors:** Philipp Warnke, Annette Devide, Mirjam Weise, Hagen Frickmann, Norbert Georg Schwarz, Holger Schäffler, Peter Ottl, Andreas Podbielski

**Affiliations:** 1 Institute of Medical Microbiology, Virology, and Hygiene, University Medicine Rostock, Rostock, Germany; 2 Department of Tropical Medicine at the Bernhard Nocht Institute, German Armed Forces Hospital of Hamburg, Hamburg, Germany; 3 Infectious Disease Epidemiology, Bernhard Nocht Institute for Tropical Medicine, Hamburg, Germany; 4 Department of Internal Medicine, Division of Gastroenterology and Endocrinology, University Medicine Rostock, Rostock, Germany; 5 Department of Prosthodontics and Material Sciences, University Medicine Rostock, Rostock, Germany; Purdue University, UNITED STATES

## Abstract

This study investigates the quantitative bacterial recovery of Methicillin-resistant S*taphylococcus aureus* (MRSA) in nasal screenings by utilizing dry or moistened swabs within an *in vivo* and an *in vitro* experimental setting. 135 nasal MRSA carriers were each swabbed in one nostril with a dry and in the other one with a moistened rayon swab. Quantitative bacterial recovery was measured by standard viable count techniques. Furthermore, an anatomically correct artificial nose model was inoculated with a numerically defined suspension of MRSA and swabbed with dry and moistened rayon, polyurethane-foam and nylon-flocked swabs to test these different settings and swab-materials under identical laboratory conditions. *In vivo*, quantities of MRSA per nostril in carriers varied between <10^1^ and >10^7^ colony forming units, with a median of 2.15x10^4^ CFU. However, no statistically significant differences could be detected for the recovery of MRSA quantities when swabbing nasal carriers with moist or dry rayon swabs. *In vitro* testing confirmed the *in vivo* data for swabs with rayon, polyurethane and nylon-flocked tips, since pre-moistening of swabs did not significantly affect the quantities of retrieved bacteria. Therefore, pre-moistening of swabs prior to nasal MRSA sampling provides no advantage in terms of recovering greater bacterial quantities and therefore can be omitted. In addition, this situation can be mimicked in an *in vitro* model, thereby providing a useful basis for future *in vitro* testings of new swab types or target organisms for screening approaches.

## Introduction

Nasal carriage of *S*. *aureus* is present in 20–30% of the population [1;2] and is a major risk factor for multiple types of purulent endogenous infections as well as bacterial transmission both in private and nosocomial environments [3–9]. As *S*. *aureus* predominantly colonizes the anterior part of the nasal cavity [10] swab based screening is commonly used to identify nasal carriers.

Different swab-types and transport systems strongly vary with regard to the uptake and release capacities of liquid and bacteria [11–19]. Additionally, utilizing the correct swabbing technique can significantly improve the bacterial recovery rate even when employing the same swab-type, for example in nasal MRSA screenings [20]. As patients benefit from fast diagnostics and therapies based on such results [21–25] it seems prudent to use the best sampling devices and techniques in order to recover the highest bacterial amounts when analyzing the sample in the laboratory. Otherwise the target microorganism could be completely missed or, at least, valuable time will be lost if augmentation in liquid broth or subcultures becomes necessary.

In daily clinical routine medical staff members collect samples with dry or moistened swab tips mainly based on individual beliefs rather than on evidence, as distinct recommendations for patient sampling are lacking. In addition, studies on the efficacy of pre-moistening swab-tips are mainly based on sampling from inert environmental surfaces [26–28].

To address the question of pre-moistening swab-tips, the present study analyzed the bacterial quantities recovered from patient samples that were collected from the nose either with a moistened or a dry rayon swab. Furthermore, the suitability of a recently introduced, anatomically correct, artificial nose model [29;30] for future standardized tests of new swab types and sampling techniques while avoiding bias due to inter-individual differences concerning anatomy, epithelial moisture, or bacterial densities in patients’ noses, was extended. This model was inoculated with a defined amount of MRSA and subsequently used to analyze bacterial recovery rates from different swab-types under identical laboratory conditions. Thus, *in vivo* and *in vitro* data of swab pre-moistening in MRSA screening are synoptically combined.

## Material and Methods

### Study design

For *in vivo* experiments, subjects were swabbed in one nostril with a dry and in the other one with a moistened rayon swab. Recovered MRSA quantities were assessed. In total, n = 135 participants (males, n = 78; females, n = 57) were recruited from the tertiary hospital of the University Medicine Rostock. Inclusion criteria were (i) a positive result in a routineously performed nasal MRSA screening diagnosed by the accredited diagnostic laboratory of University Medicine Rostock; (ii) no ongoing decolonization procedure; (iii) no antibiotic treatment. To avoid bias due to sample collection order, for instance by always targeting the left nostril with a dry and the right nostril with a moistened swab, the collection mode was altered after swabbing a series of ten patients.

For *in vitro* experiments, artificial, autoclave resistant, silicone nose models, based on an imprint of a human nose [29], were inoculated with a numerically defined amount of MRSA and subsequently swabbed. Four different swab types were tested. Nose models were swabbed in one nostril with a dry and in the other one with a moistened swab. For each swab type and swabbing scenario n = 10 swabs were tested. Recovered MRSA quantities were assessed.

### Swabs

1. Rayon: Nerbe Plus, Winsen/Luhe, Germany, Invasive sterile collection swab, ref. 09-812-8051
2. Polyurethane cellular foam: MWE medical wire, Corsham Wiltshire England, Sigma Dry Swab Tubed, ∑-Swab, ref. MW941
3. Nylon flocked fiber: Copan, Brescia, Italy, FLOQSwabs, regular, ref 552C
4. Rayon: MWE medical wire, Corsham Wiltshire England, Tubed Sterile Dryswab, ref. MW102

Swabbing of patients was exclusively performed with Nerbe Plus (Invasive sterile collection swab), swabbing of the artificial nose model was executed with all four swab types.

### Bacterial culture techniques for *in vitro* experiments

*Staphylococcus aureus* MRSA strain (ST22-MRSA-IV, Barnim epidemic strain) and *Staphylococcus epidermidis* strain (DSMZ 1798) were separately propagated at 37°C in brain-heart-infusion (BHI) medium as overnight standing cultures in ambient air. Early stationary phase cells were harvested, washed in phosphate-buffered saline (PBS; NaCl (137 mmol/l), KCl (2.7 mmol/l), Na_2_HPO_4_ x 2 H_2_O (10 mmol/l), KH_2_PO_4_ (2.0 mmol/l)) at pH 7.4 and resuspended in BHI + 20% glycerol. Aliquots were stored at -80°C for up to 3 months. Each stock concentration was determined by viable cell count of 3 representative tubes according to standard techniques.

### Inoculation of the nose models

Nose models were prepared at the day of usage. Autoclaved, sterile nose models were inoculated with a suspension of MRSA and *Staphylococcus epidermidis* strains following a protocol as described elsewhere [20]. Briefly, amounts of 2x10^4^ colony forming units (CFU) MRSA (adjusted to the median bacterial quantities recovered from patients) and 1x10^5^ CFU *Staphylococcus epidermidis* (to simulate nasal flora) per nostril were applied at defined locations in each nasal vestibule, distributed in two 10 μl droplets of bacterial suspension. Inoculated nose models were dried for one hour at room temperature.

Inoculation dose was controlled by plating serial dilutions of the bacterial suspension onto Columbia agar supplemented with 5% sheep blood (BD, Heidelberg, Germany) followed by cultivation at 37°C under ambient atmosphere for 48 h and CFU counting.

### Sample collection techniques

Subjects and nose models were swabbed according to a protocol as described elsewhere [20]. Briefly, for both situations one swab was used per nostril. Swabs were either used in dry state directly from the sterile sleeve or pre-moistened by dipping the swab tip into a sterile 15 ml Round Bottom Tube (Greiner Bio-One, Frickenhausen, Germany, Ref. 164161) containing 1 ml of 0.85% NaCl solution, followed by thoroughly squeezing at the tube wall to discard surplus liquid before usage. The nasal vestibule was swabbed by applying gentle pressure. The swab was rotated while circulating in the nasal vestibule for approximately 5 seconds.

After swabbing the nostrils, moist swabs were placed in the corresponding tube used to moisten the swab. In the case of dry swabs, these were set down into a fresh sterile 15 ml Round Bottom Tube containing 1 ml of sterile 0.85% NaCl solution.

### Quantification of bacteria

All swabs were subjected to microbiological analysis immediately after swabbing the noses or the nose models. The swab-NaCl-combination was vortexed for 5 seconds. CFU were determined by plating 100 μl of 1:10 serial dilutions onto Columbia agar supplemented with 5% sheep blood (BD, Heidelberg, Germany), resulting in a detection threshold of 10 CFU. Bacterial amounts below this limit could not be detected and swabs were regarded as MRSA negative. In parallel, a chromogenic MRSA medium (chromID MRSA, bioMérieux, Marcy l’Etoile, France) was inoculated with 100 μl of the undiluted suspension. Agar plates were subsequently cultured at 37°C under ambient atmosphere for 48 h. CFU were then counted by macroscopic inspection. *Staphylococcus aureus* was identified by β-hemolysis and colony color (golden yellow on Columbia agar, green color on chromID MRSA agar), if necessary by agglutination assay (Slidex Staph Plus, bioMérieux, Marcy l’Etoile, France), or by matrix-assisted laser-desorption-ionization time-of-flight mass spectrometry (MALDI-TOF-MS) using a Shimadzu “AXIMA Assurance” MALDI-TOF mass spectrometer (Shimadzu Germany Ltd., Duisburg, Germany) as described elsewhere [31].

### Statistical analysis

Data were analyzed using nonparametric Wilcoxon-Mann-Whitney U-test. All p-values resulted from two-tailed statistical testing. p-values of <0.05 were considered to be statistically significant. Raw data of *in vivo* and *in vitro* experiments are provided in the supplementary information (S1 Table, S2 Table).

### Ethics statement

All clinical investigation has been conducted according to the principles expressed in the Declaration of Helsinki. The study was approved by the institutional review board of University Medicine Rostock (Ethikkommission an der Medizinischen Fakultät der Universität Rostock; approval no: A 2012–0079) in line with national and ICH-GCP guidelines. All patients provided written informed consent.

## Results

### Recovered MRSA quantities from patients utilizing dry or moist rayon swabs

**Fig 1:**
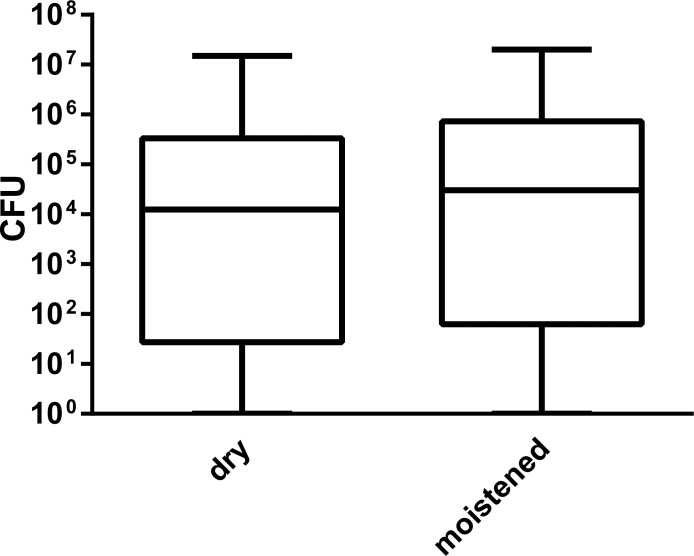
Recovered MRSA quantities from patients utilizing dry or moistened rayon swabs. Patients were swabbed in the nostrils utilizing either dry or moist rayon swabs. CFU = colony forming units. Data result from n = 135 patients. p-value = 0.17 (Wilcoxon-Mann-Whitney U-test).

Patients were swabbed with dry or moist rayon swabs. The median of the recovered bacterial quantities obtained with dry nasal swabs was log_10_ CFU = 4,10 (1.25x10^4^ CFU), with moistened swabs log_10_ CFU = 4,48 (median = 3.05x10^4^ CFU). These differences were not statistically significant (p = 0.17) (Fig 1).

### Recovered MRSA quantities from the artificial nose model utilizing dry or moist rayon, polyurethane-foam and nylon-flocked swabs

**Fig 2:**
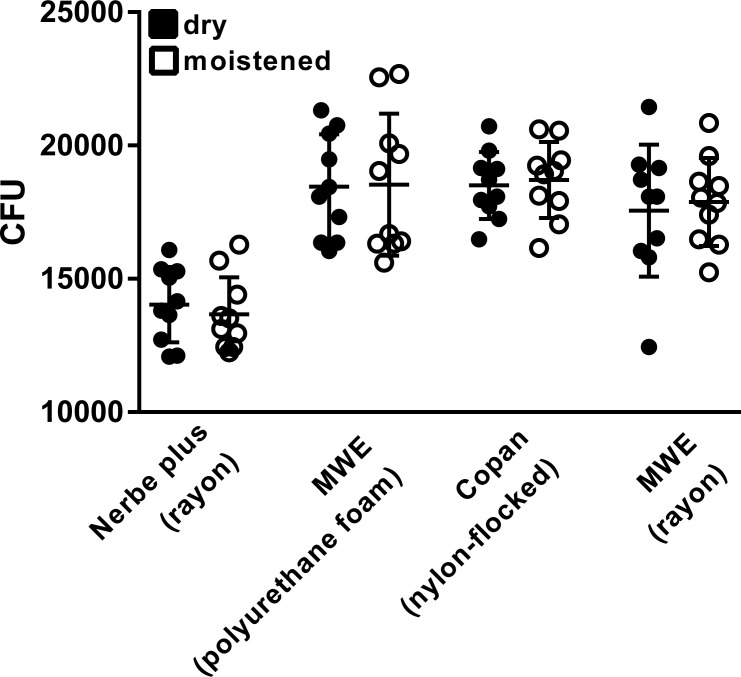
Recovered MRSA quantities from the artificial nose model utilizing dry or moist rayon, polyurethane foam and nylon-flocked swabs. Nose models were swabbed utilizing either dry or moistened rayon, polyurethane foam or nylon-flocked swabs. Data results from n = 10 swabs for each setting. p-value (comparing dry and moist swabs within one swab-type) = 0.62 (Nerbe plus, rayon); 0.90 (MWE, polyurethane); 0.72 (Copan, nylon-flocked); 0.96 (MWE, rayon) (Wilcoxon-Mann-Whitney U-test).

Inoculated nose models were tested with dry or moistened swabs of the indicated swab-types. Bacterial quantities were assessed. Within the same swab-type, no statistically significant differences could be detected between dry or moist sample collection (p = 0.62 (Nerbe plus, rayon); 0.90 (MWE, polyurethane); 0.72 (Copan, nylon-flocked); 0.96 (MWE, rayon)) (Fig 2).

Statistically significant differences could be detected when comparing recovered bacterial quantities between different swab-types. From MWE polyurethane, Copan nylon-flocked and MWE rayon swabs statistically significant greater quantities of MRSA could be recovered in comparison to Nerbe plus rayon swabs in the dry (p<0.0001; p<0.0001; p = 0.0014, respectively) as well as in the moist (p<0.0001; p<0.0001; p<0.0001, respectively) setting.

Swabs with tips made of PU-foam and nylon-flocked fibers performed best in terms of recovering the greatest bacterial quantities. Performance between these swabs types was similar with no statistically significant differences in both swabbing settings (p>0.05).

MWE rayon swabs recovered less bacterial quantities as compared to the PU foam or nylon-flocked swabs, but these differences were not statistically significant (p>0.05).

### Nasal MRSA burden in patients

For this analysis, data from both the dry and moist setting were used, i.e. about half the results per nostril were obtained by sample collection with either dry or moistened swabs. Quantities of MRSA per nostril were clustered in log_10_ steps. Bacterial quantities ranged between <10^1^ and >10^7^ CFU (Table 1).

**Table 1:** Quantities of MRSA per nostril.

Quantities of MRSA per nostril								
CFU	<10^1^	10^1^−10^2^	10^2^−10^3^	10^3^−10^4^	10^4^−10^5^	10^5^−10^6^	10^6^−10^7^	>10^7^
***n*** (= 270)	56	21	27	13	54	46	50	3

Data from moist and dry swabs are included. CFU = colony forming units; MRSA = Methicillin resistant *Staphylococcus aureus*.

First, several patients that were initially tested as MRSA-positive and were therefore included in this study, now presented themselves as MRSA-negative (see also next subchapter). Second, based upon the documented MRSA-quantities, the MRSA-positive patient population segregated into two major groups, one with less than 1,000 MRSA CFU per nostril and another even larger one with 10,000 to 1,000,000 MRSA CFU per nostril.

### Sensitivity of dry or moist rayon swabs for the detection of MRSA

All patients were initially screened positive for carriage of MRSA in the routine MRSA screening program of the hospital. The study population was therefore stated as 100% MRSA positive. Under this aspect, sensitivity of consecutively executed sample collection with dry or moist nasal swabs was qualitatively analyzed. Both sampling methods displayed similar sensitivities. By sampling with dry rayon swabs 79.1% of the initially positive tested patients were MRSA positive, with moistened rayon swabs 79.4%, respectively.

## Discussion

In daily hospital routine swab-based nasal screening is commonly used to identify nasal carriers of MRSA. As laboratory MRSA analyses based upon solid and liquid culture media or PCR depend on the bacterial uptake and release capacities of the swabs, optimum preanalytical conditions are crucial.

Prior studies had shown that the recovery of different bacteria from environmental surfaces could be augmented if pre-moistened swabs were used [26–28]. Regarding the optimal method to detect *S*. *aureus* on environmental surfaces and fomites the literature is inconsistent [32–36]. Some study protocols use moistened swab based sampling for medical devices [37] or for sampling of animals [38] or to augment the DNA yield from touch samples or saliva in forensic medicine [39–41]. However, for detection of group A streptococci by throat swabs, better performance of dry swabs was demonstrated [42], indicating that pre-moistening is not always the high road to increase swabbing sensitivity rates.

The present study analyzed the impact of pre-moistened or dry swabs on the quantitative recovery of MRSA in an *in vivo* and *in vitro* approach. By swabbing patients with dry or moist rayon swabs, it was clearly demonstrated, that this procedure had no impact on the laboratory result. Recovered MRSA quantities were similar in both approaches. This is in line with previous studies comparing dry or moist swabs in nasal MRSA screening [43;44]. However, these latter studies had no distinct sampling protocol and only very small sample sizes of 8 and 29 MRSA patients, respectively, which indicates that these studies could have been underpowered. The present study confirmed these results with a substantial number of patients. Furthermore, a strict sampling protocol [20] was employed providing a profound basis for the obtained results.

Swabbing of patients previously identified as MRSA carriers with pre-moistened or dry rayon swabs resulted in comparable MRSA detection sensitivities of approximately 80%, illustrating that both sampling procedures are similar even on a qualitative level. The discrepancy of the re-test result to the initial MRSA screening result could be due to an intermittent versus a persistent carrier status of *S*. *aureus* [1;45;46], to a nasal MRSA burden close to the detection limit, or to the usage of rayon swabs that trap a share of the initially absorbed bacteria and consecutively, hamper their release [47].

The nasal burden found in this study varies within a CFU range of seven log_10_ steps. This is well in the range of another study that investigated 3–15,000,000 CFU of MRSA in the nares per swab [48]. This heterogeneous composition of nasal MRSA burden was also described in semiquantitative cultural analyses [49] or PCR based cycle threshold (C_t_) quantification [50]. But all of the quantitative studies had to be considered with caution, as laboratory detection significantly depends on the swab type and sampling conditions applied.

In addition, in the present study sampling with moistened and dry swabs was assessed under identical laboratory conditions utilizing an anatomically correct artificial model of a human nose [29]. Recently, this model had successfully been used for device testing and teaching of medical staff in terms of MRSA sampling [20;30]. For inoculation *S*. *aureus*, ST22-MRSA-IV strain, i.e. the Barnim epidemic strain, was chosen, since it is the predominant MRSA strain in many countries [51–58]. The inoculation dose was adapted to the median MRSA quantity that was recovered from the *in vivo* swabbing approach of this study. Rayon, polyurethane foam and nylon-flocked swabs were tested, as these swab types represent the most common swab-tip materials and were demonstrated to perform different within different sampling conditions [19]. Again, using these swab types in dry or moist setting did not result in different recovered bacterial quantities.

This study is limited by the fact, that an influence of different transport media was not assessed. Since transport media are used to prevent overgrowth by contaminants or reduced cultivation rates of pathogens, this aspect could be negligible, because transport and processing of the swabs were simulated under optimum conditions. The semiquantitative processing of swabs, i.e. streaking the swab tip over the surface of an agar plate, which is still daily routine in some laboratories, was not investigated, as the present study focused on quantitative analyses. But prior studies analyzing release capacities of swabs utilizing this streak technique indicated a similar performance on a lower quantitative recovery level [19;30]. Further processes interfering with culture-based analyses could be circumvented when applying PCR based screening techniques.

In conclusion, using dry or moist swabs for nasal MRSA screening does not affect the amount of the recovered bacteria in our *in vivo* and *in vitro* setting. Furthermore, sampling with dry swabs needs less manual handling. Therefore, dry sampling appears to be the mode of choice.

## Supporting Information

S1 Table Raw data of *in vivo* experiments. Recovered bacterial quantities in CFU from patient sample collection with dry or moistened Nerbe plus rayon swabs.
(DOCX)

S2 Table Raw data of *in vitro* experiments. Recovered bacterial quantities in CFU from sample collection with dry or moistened swabs in the artificial nose model.
(DOCX)
