# Supplementary material for: Utilizing Moist or Dry Swabs for the Sampling of Nasal MRSA Carriers? An In Vivo and In Vitro Study
Source: PLoS One. 2016 Sep 14;11(9):e0163073. doi: 10.1371/journal.pone.0163073 (PMC5023121; doi:10.1371/journal.pone.0163073)
Supplement: S1 Table — Recovered bacterial quantities in CFU from patient sample collection with dry or moistened Nerbe plus rayon swabs. (DOCX) [file pone.0163073.s001.docx]

**S1 Table. Raw data of *in vivo* experiments.**

Recovered bacterial quantities in CFU from patient sample collection with dry or moistened Nerbe plus rayon swabs.

| **dry** | | | | |
| --- | --- | --- | --- | --- |
| 300 | 37000 | 1200000 | 40 | 0 |
| 2100 | 14000 | 430000 | 280 | 0 |
| 400 | 26000 | 3270000 | 280 | 0 |
| 3900 | 3000000 | 3500000 | 30 | 0 |
| 700 | 120000 | 740000 | 40 | 0 |
| 300 | 300000 | 3000000 | 70 | 0 |
| 3200 | 15000000 | 180000 | 10 | 0 |
| 6300 | 20000 | 4000000 | 20 | 0 |
| 2500 | 230000 | 1550000 | 120 | 0 |
| 5700 | 1030000 | 920000 | 20 | 0 |
| 12000 | 730000 | 110000 | 140 | 0 |
| 27000 | 2300000 | 30000 | 70 | 0 |
| 13000 | 1350000 | 3000000 | 20 | 0 |
| 33000 | 370000 | 1430000 | 120 | 0 |
| 27000 | 760000 | 1500000 | 170 |  |
| 26000 | 2200000 | 170000 | 160 |  |
| 12000 | 1440000 | 1340000 | 0 |  |
| 34000 | 160000 | 30000 | 0 |  |
| 49000 | 4480000 | 200000 | 0 |  |
| 39000 | 530000 | 5000000 | 0 |  |
| 15000 | 90000 | 600000 | 0 |  |
| 10000 | 70000 | 410 | 0 |  |
| 19000 | 320000 | 70 | 0 |  |
| 85000 | 2500000 | 110 | 0 |  |
| 21000 | 2000000 | 10 | 0 |  |
| 10000 | 690000 | 80 | 0 |  |
| 87000 | 1070000 | 370 | 0 |  |
| 39000 | 130000 | 80 | 0 |  |
| 83000 | 2170000 | 170 | 0 |  |
| 42000 | 370000 | 110 | 0 |  |
| **moistened** | | | | |
| 3700 | 3800000 | 3000000 | 60 | 0 |
| 7300 | 6000000 | 1200000 | 70 | 0 |
| 5300 | 4000000 | 550000 | 110 | 0 |
| 5800 | 12000000 | 340000 | 10 | 0 |
| 4100 | 20000 | 740000 | 250 | 0 |
| 1800 | 1500000 | 240000 | 90 | 0 |
| 34000 | 750000 | 110000 | 670 | 0 |
| 38000 | 3300000 | 1800000 | 130 | 0 |
| 18000 | 830000 | 1800000 | 170 | 0 |
| 61000 | 3200000 | 1020000 | 50 | 0 |
| 12000 | 1000000 | 150000 | 40 | 0 |
| 26000 | 290000 | 90000 | 570 | 0 |
| 54000 | 350000 | 120000 | 20 | 0 |
| 22000 | 1090000 | 530000 | 270 | 0 |
| 8000 | 1650000 | 360000 | 230 | 0 |
| 87000 | 1800000 | 4400000 | 30 | 0 |
| 42000 | 110000 | 2500000 | 240 |  |
| 19000 | 2040000 | 4880000 | 200 |  |
| 16000 | 20000000 | 3000000 | 0 |  |
| 14000 | 190000 | 2500000 | 0 |  |
| 27000 | 50000 | 130000 | 0 |  |
| 54000 | 2140000 | 730000 | 0 |  |
| 39000 | 590000 | 720000 | 0 |  |
| 31000 | 290000 | 5000000 | 0 |  |
| 30000 | 200000 | 320000 | 0 |  |
| 59000 | 310000 | 4790000 | 0 |  |
| 25000 | 2000000 | 470000 | 0 |  |
| 28000 | 1700000 | 5000000 | 0 |  |
| 21000 | 110000 | 310000 | 0 |  |
| 10000 | 1400000 | 160 | 0 |  |
