# Supplementary material for: Utilizing Moist or Dry Swabs for the Sampling of Nasal MRSA Carriers? An In Vivo and In Vitro Study
Source: PLoS One. 2016 Sep 14;11(9):e0163073. doi: 10.1371/journal.pone.0163073 (PMC5023121; doi:10.1371/journal.pone.0163073)
Supplement: S2 Table — Recovered bacterial quantities in CFU from sample collection with dry or moistened swabs in the artificial nose model. (DOCX) [file pone.0163073.s002.docx]

**S2 Table. Raw data of *in vitro* experiments.**

Recovered bacterial quantities in CFU from sample collection with dry or moistened swabs in the artificial nose model.

| **dry** | | | | **moistened** | | | |
| --- | --- | --- | --- | --- | --- | --- | --- |
| **Nerbe plus, rayon** | **MWE, PU foam** | **Copan, nylon-flocked** | **MWE, rayon** | **Nerbe plus, rayon** | **MWE, PU foam** | **Copan, nylon-flocked** | **MWE, rayon** |
| 12120 | 16360 | 18720 | 12440 | 13600 | 15600 | 17920 | 18040 |
| 13800 | 17320 | 20720 | 19160 | 16280 | 22560 | 16160 | 16480 |
| 15040 | 20440 | 18080 | 21440 | 12960 | 16320 | 20600 | 15240 |
| 13640 | 18080 | 16480 | 18080 | 12440 | 16320 | 19440 | 18640 |
| 12720 | 19480 | 19120 | 19280 | 12240 | 22680 | 20560 | 20840 |
| 15280 | 16040 | 17720 | 18080 | 13120 | 19680 | 18920 | 17840 |
| 12080 | 21320 | 19160 | 16520 | 14400 | 16680 | 17040 | 19600 |
| 14160 | 18440 | 19800 | 15800 | 12440 | 20080 | 19240 | 16280 |
| 15360 | 16360 | 17960 | 18720 | 15680 | 16400 | 18120 | 17400 |
| 16080 | 20760 | 17240 | 16040 | 13520 | 19040 | 19040 | 18480 |
